# Chiral-Dependent Redox Capacitive Biosensor Using Cu-Cys-GSH Nanoparticles for Ultrasensitive H_2_O_2_ Detection

**DOI:** 10.3390/bios15050315

**Published:** 2025-05-14

**Authors:** Duygu Yilmaz Aydin, Jie Jayne Wu, Jiangang Chen

**Affiliations:** 1 Department of Electrical Engineering and Computer Science, The University of Tennessee, Knoxville, TN 37996, USA; 2 Department of Bioengineering, Malatya Turgut Ozal University, 44210 Malatya, Türkiye; duygu.aydin@ozal.edu.tr; 3 Department of Public Health, The University of Tennessee, Knoxville, TN 37996, USA; jchen38@utk.edu

**Keywords:** AC electrokinetics (ACEK), capacitive sensors, Fenton-like reaction, hydrogen peroxide (H_2_O_2_) detection, Cu-Cys-GSH nanoparticles

## Abstract

Copper-thiolate nanostructures, formed through the self-assembly of cysteine (Cys) and glutathione (GSH) with copper ions, offer a versatile platform for redox-active applications due to their structural stability and chemical functionality. In this study, Cu-Cys-GSH nanoparticles were synthesized and employed to develop a capacitive biosensor for the ultralow concentration detection of hydrogen peroxide (H_2_O_2_). The detection mechanism leverages a Fenton-like reaction, where H_2_O_2_ interacts with Cu-Cys-GSH nanoparticles to generate hydroxyl radicals (·OH) through redox cycling between Cu^2+^ and Cu^+^ ions. These redox processes induce changes in the sensor’s surface charge and dielectric properties, enabling highly sensitive capacitive sensing at gold interdigitated electrodes (IDEs). The influence of chirality on sensing performance was investigated by synthesizing nanoparticles with both L- and D-cysteine enantiomers. Comparative analysis revealed that the stereochemistry of cysteine impacts the catalytic activity and sensor response, with Cu-L-Cys-GSH nanoparticles exhibiting superior performance. Specifically, the biosensor achieved a linear detection range from 1.0 fM to 1.0 pM and demonstrated an ultra-sensitive detection limit of 21.8 aM, outperforming many existing methods for H_2_O_2_ detection. The sensor’s practical performance was further validated using milk and saliva samples, yielding high recovery rates and confirming its robustness and accuracy for real-world applications. This study offers a disposable, low-cost sensing platform compatible with sustainable healthcare practices and facilitates easy integration into point-of-care diagnostic systems.

## 1. Introduction

Hydrogen peroxide (H_2_O_2_) is a crucial biomolecule involved in various physiological and pathological processes, particularly in oxidative stress, immune response, and inflammation [1,2]. As a key reactive oxygen species (ROS), abnormal levels of H_2_O_2_ are closely associated with the progression of chronic diseases, including cancer, neurodegenerative disorders, and cardiovascular conditions [3,4,5,6,7,8]. Therefore, the development of highly sensitive and selective detection methods for H_2_O_2_ in biological samples is essential for early disease diagnosis, clinical monitoring, and biomedical research.

Nanomaterials have gained significant attention as biosensing platforms due to their unique physicochemical properties, including high surface area, tunable electronic states, and excellent catalytic activity [9,10,11,12,13,14,15,16,17,18]. The integration of nanomaterials into biosensors has significantly improved their analytical performance and utilization in the biomedical field. In the context of H_2_O_2_ detection, nanoparticles such as gold, silver, platinum, iron oxide, rhodium, nickel oxide, copper, and bimetallic nanoparticles have been widely used to enhance sensor performance [19,20,21]. Silver nanoparticles deposited on reduced graphene oxide and cerium(IV) oxide nanocomposites have been employed for nonenzymatic electrochemical detection of H_2_O_2_, demonstrating high sensitivity and stability [22]. Similarly, platinum-doped graphene sheets combined with cerium(IV) oxide have been integrated into screen-printed electrodes for selective H_2_O_2_ sensing, showcasing improved electron transfer and catalytic activity [23]. Additionally, Fe_3_O_4_-Au magnetic nanoparticles have been utilized to immobilize horseradish peroxidase on graphene-based electrodes, enabling sensitive electrochemical H_2_O_2_ biosensing [24]. Rhodium nanoparticles have been employed to modify graphite electrodes, facilitating the electrochemical detection of H_2_O_2_ even in the presence of oxygen and from tea extracts, highlighting their robust catalytic properties [25]. Furthermore, platinum-modified multi-walled carbon nanotube electrodes have been fabricated for rapid and quantitative H_2_O_2_ detection, particularly in food safety applications with high sensitivity and reproducibility [26]. Copper-based nanoparticles, particularly those incorporating cysteine and glutathione ligands, have attracted interest due to their redox activity and potential role in catalytic processes. Studies have shown that Cu-Cys nanoparticles exhibit self-assembly behavior and can engage in redox cycling between Cu^2+^ and Cu^+^, making them suitable for reactions involving H_2_O_2_ [27]. The addition of glutathione to form Cu-Cys-GSH nanoparticles further enhances their stability and reactivity, which has been explored in contexts such as chemodynamic therapy and oxidative stress modulation. The interaction of Cu-Cys-GSH nanoparticles with H_2_O_2_ leads to a redox cycling process where Cu^2+^ is reduced to Cu^+^ in the presence of glutathione, followed by the Cu^+^-catalyzed decomposition of H_2_O_2_ to produce highly reactive hydroxyl radicals (·OH) [28]. This mechanism mimics the classical Fenton reaction observed with iron but operates more efficiently at neutral or slightly acidic pH, making it highly suitable for biosensing applications [29]. Recent studies have demonstrated that Cu-Cys-GSH nanoparticles exhibit peroxidase-mimicking activity, catalyzing the oxidation of substrates such as 3,3′,5,5′-tetramethylbenzidine (TMB) in the presence of H_2_O_2_, confirming their role in ROS generation and redox cycling. Specifically, the oxidation of TMB to its oxidized form (oxTMB) was observed through a significant increase in UV–vis absorption, whereas the presence of GSH reversed this process by reducing oxTMB back to TMB, leading to a measurable decrease in absorption [30].

Although chromatography, colorimetry, chemiluminescence, and fluorometry are widely used for H_2_O_2_ detection, they are often limited by background interference, limited sensitivity thresholds, high operational costs, and the requirement for complex, specialized instrumentation. Additionally, many approaches rely heavily on labeling strategies, which can further complicate procedures and hinder real-time analysis. Capacitive biosensors have emerged as a promising alternative, offering label-free, real-time monitoring of molecular interactions based on changes in dielectric properties and surface charge distribution at the electrode interface. These sensors rely on variations in capacitance caused by biomolecular interactions, making them highly sensitive for detecting ultralow concentrations of analytes. Additionally, recent advances in AC electrokinetics (ACEK) effects have demonstrated the potential to further enhance capacitive sensing by actively directing biomolecules toward the electrode surface, overcoming the limitations of passive diffusion [31,32,33,34]. The ACEK effect, including AC electrothermal (ACET) flow, enables long-range transport of analytes, ensuring effective interaction with the sensor surface, thereby accelerating detection speed and improving sensitivity [35].

The integration of Cu-Cys-GSH nanoparticles with ACEK-assisted capacitive biosensing provides a novel and highly efficient strategy for H_2_O_2_ detection. The catalytic activity of these nanoparticles, driven by the Fenton-like reaction, induces dynamic changes in surface charge and dielectric properties upon H_2_O_2_ interaction. This process enhances the capacitive signal, enabling real-time and highly selective detection at femtomolar concentrations. Additionally, the stereochemistry of cysteine in Cu-Cys-GSH nanoparticles influences catalytic efficiency, further affecting sensor performance [29].

**Figure 1:**
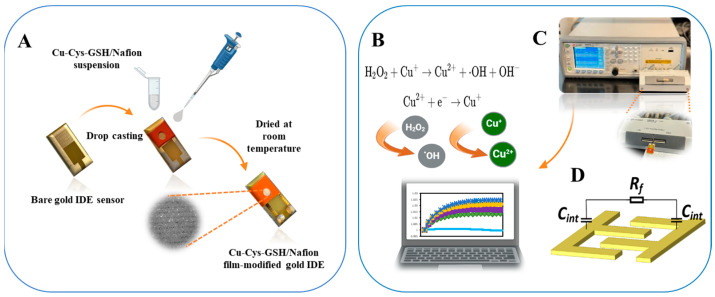
Schematic diagram of (**A**) functionalization of the gold-IDE sensor with Cu-Cys-GSH/Nafion film, (**B**) Fenton-like reaction mechanism involved in detection, (**C**) measurement setup for ACEK-capacitive sensing, where interfacial capacitance is acquired at a fixed voltage through an LCR meter and transferred to a computer for processing, and (**D**) equivalent circuit model representing interfacial capacitance.

This study aims to develop a Cu-Cys-GSH nanoparticle–based capacitive biosensor for ultra-trace H_2_O_2_ detection while investigating the chiral effects on sensor performance. The detection mechanism leverages a Fenton-like reaction, wherein Cu^2+^ and Cu^+^ ions undergo redox cycling in the presence of H_2_O_2_, generating hydroxyl radicals (·OH) (Figure 1B). This process alters the interfacial capacitance at the gold interdigitated electrode surface, enabling highly sensitive detection. Furthermore, investigating the influence of nanoparticle chirality on sensor performance provides insights into the role of stereochemistry in catalytic efficiency and signal transduction. The applicability of the biosensor under real-world conditions is validated using milk samples. This work demonstrates the potential of Cu-Cys-GSH nanoparticles in capacitive biosensing, offering a low-cost, disposable platform for real-world applications in point-of-care testing.

## 2. Materials and Methods

### 2.1. Reagents and Apparatus

Copper(II) chloride dihydrate (CuCl_2_·2H_2_O) (˃99%), sodium hydroxide (NaOH), L-cysteine (˃98%), D-cysteine (98%), L-alanine (99%), L-aspartic acid (99%), dopamine hydrochloride (99%), phosphate-buffered saline (PBS, 10X) at pH 7.4 were purchased from Thermo Scientific (Waltham, MA, USA). Acetone, isopropyl alcohol (IPA), and Nafion were supplied by Sigma-Aldrich (Darmstadt, Germany). L-glutathione (GSH) reduced was bought from Cayman Chemical (Ann Arbor, MI, USA). Testing buffer 0.1X PBS pH 7.4 was prepared by diluting 10X PBS with Milli-Q water.

The morphologies of the sensor and chiral nanoparticles were characterized using scanning electron microscopy (SEM TM-4000Plus, Hitachi, Tokyo, Japan). X-ray diffraction (XRD) patterns of nanoparticles were recorded on a Panalytical Empyrean q-2q diffractometer (Malvern Panalytical, Malvern, UK). Sufficient counting statistics were achieved using a 0.02626° 2q step scan from 3 to 70°, with an exposure time of 114.75 s per step and a revolution spin rate of 2 s. Fourier transform infrared spectra (FTIR) were obtained on a Thermo Scientific FTIR spectrometer (Waltham, MA, USA). A precision LCR meter (Keysight^®^ E4980A, Santa Rosa, CA, USA) was utilized for electrical measurements.

### 2.2. Preparation of Cu-Cys-GSH Nanoparticles

NaOH (2 mmol) and either L- or D-cysteine (2 mmol) were dissolved in deionized water and slowly added dropwise into an aqueous CuCl_2_ solution under stirring for 5 min to facilitate the formation of Cu-Cys nanoparticles. The resulting Cu(II)-Cys nanoparticles were then separated via centrifugation and washed sequentially with deionized water and alcohol. This synthesis method was first introduced in [28].

The Cu(II)-Cys nanoparticles were mixed with 50 mM L-GSH under a nitrogen atmosphere and stirred for 2 h. The resulting Cu-Cys-GSH nanoparticles were collected via centrifugation and washed with deionized water [30].

### 2.3. Preparation of Cu-Cys-GSH Nanoparticle–Functionalized Gold IDE Sensor

The preparation of the gold IDEs with a metallic trilayer structure (Cu/Ni/Au) involved sequential surface treatment, electroplating, and cleaning. The width of the IDE finger and the gap between adjacent fingers are 100 μm. Initially, the electrodes underwent mechanical polishing using polishing pads to achieve a smooth and uniform surface. Subsequently, a thorough cleaning procedure was performed by sequentially rinsing the electrodes with acetone, isopropanol (IPA), and Milli-Q water, followed by air drying to eliminate any residual contaminants. A thin gold layer was electrodeposited onto the surface using a two-electrode configuration. Following electroplating, the electrodes underwent an additional cleaning cycle using acetone, IPA, and Milli-Q water, followed by air drying. To enhance surface hydrophilicity, the gold IDEs were subjected to UV–ozone treatment for 25 min. Finally, a 2 mm-thick silicone chamber was affixed to the electrode surface to secure the samples for subsequent experimental procedures.

A total of 15 mg of Cu-Cys-GSH nanoparticles was dispersed in 1 mL of 0.5% Nafion solution to form a homogenous dispersion under vigorous ultrasonication for about 1 h. Then, 8 µL of the resulting Cu-Cys-GSH/Nafion dispersion was dropped onto the surface of gold IDEs and was kept at room temperature till dry (Figure 1A).

### 2.4. Sample Preparation

To evaluate the applicability of the developed biosensor, H_2_O_2_ detection was performed in milk. Milk samples, purchased from a local market, were centrifuged at 10,000 rpm for 10 min to sediment fats, proteins, and other macromolecular components [36]. The clear supernatant was collected and diluted 10-fold with 0.05X PBS. Spiked recovery experiments were conducted by introducing H_2_O_2_ at concentrations of 10 fM, 100 fM, and 1 pM into the samples before the detection.

### 2.5. Capacitance Measurement

H_2_O_2_ stock solutions were diluted with 0.05X PBS buffer to obtain the analytical samples with concentrations ranging from 1 fM to 1 pM. During the measurement process, 15 μL of H_2_O_2_ solution was introduced into the chamber of the IDE sensor, which was then linked to an LCR meter for data acquisition and waited 5 min (Figure 1C). An AC signal of 1 kHz at 300 mV was applied to the IDE, and capacitance readings were recorded continuously for 10 s. The measured capacitance values were then normalized relative to the initial capacitance, and the rate of capacitance change was computed in terms of percentage variation per minute (dC/dt expressed in %/min). Sensor repeatability and consistency were evaluated by reporting the results as the mean ± standard deviation (SD) of measurements from three independently prepared sensors. The baseline capacitance change rate was determined using blank 0.05X PBS. The 1 kHz frequency was selected based on our previous study [37]. The voltage was optimized through experimental evaluation to achieve the best sensing performance.

## 3. Sensing Mechanism

### 3.1. Interfacial Capacitance Sensing

When an electrode is immersed in an electrolyte solution, charge accumulation occurs at the electrode surface, leading to the formation of an electric double layer (EDL). This layer consists of oppositely charged counter ions near the surface, forming an interfacial capacitance [38]. When an AC signal is applied, the solid–liquid interface can be represented by an equivalent circuit, as illustrated in Figure 1D, where Rf denotes the charge transfer resistance, and Cint corresponds to the interfacial capacitance, characterizing the capacitive behavior at the electrode–electrolyte interface. The application of an AC signal (300 mV at 1 kHz) enables the measurement of capacitance changes at the interface, which are sensitive to surface modifications induced by molecular interactions. As described in our previous studies [35,39,40,41], changes in surface morphology, whether through nanoparticle deposition or biomolecular immobilization, can significantly impact both the dielectric environment and surface morphology. These surface modifications enhance the capacitive response by increasing the interfacial area available for electric double-layer formation.

The initial capacitance is described using the following equation:

$$C_{int,0}=\frac{\varepsilon A_{int}}{d},$$

where *A_int_* is the surface area of the capacitor. *ε* and *d* are the relative permittivity and thickness of the EDL, respectively. Following nanoparticle deposition and the Fenton-like reaction, interfacial capacitance (*C_int_*_,*final*_) can be expressed as follows:

$$C_{int,final}=\frac{\varepsilon A_{final}}{d},$$

where *A_final_* is the effective surface area of the final capacitor. The relative change in capacitance is given as follows:

$$\frac{∆C}{C_{int,0}}=\frac{∆A}{A_{int}}.$$

In this study, the IDE sensor with a functionalized Cu-Cys-GSH nanoassembly layer operated as a dynamic sensing platform for H_2_O_2_ detection through capacitance variation. Functionalization with Cu-Cys-GSH nanoparticles increased the surface roughness and effective area of the electrode, leading to an initial rise in *C_int_*. The subsequent introduction of H_2_O_2_ triggered a Fenton-like reaction at the modified surface, as shown in Figure 1B, further altering the interface and enhancing *C_int_* through catalytic surface restructuring.

### 3.2. ACEK Effects

AC electrokinetic (ACEK) effects encompass a range of electrokinetic phenomena that occur when an inhomogeneous AC electric field is applied to an aqueous solution. These effects include dielectrophoresis (DEP), AC electroosmosis (ACEO), and AC electrothermal (ACET) flow, each of which contributes to the movement of particles or fluid within the system [40,41]. The interplay of these mechanisms significantly influences the capacitive sensing performance of the proposed biosensor for H_2_O_2_ detection.

Dielectrophoresis (DEP) is a phenomenon in which neutral particles suspended in a solution experience motion due to polarization effects at the particle–liquid interface in response to the applied electric field gradient [42]. While DEP is a dominant force in systems involving micro- and nanoscale particles, its influence is negligible in this study because H_2_O_2_ is a small molecule that does not undergo significant polarization under the applied electric field.

AC electro-osmosis (ACEO) refers to the induced fluid flow at electrode surfaces under an inhomogeneous alternating current (AC) electric field. It plays a crucial role in microfluidic applications by enhancing mass transport and analyte mixing without external pumps. When an electrode is energized, counter ions in the electrolyte solution are attracted to the surface, forming the EDL. The inhomogeneous AC field creates a tangential electric field component, causing ion movement within the EDL. This motion generates shear stress, which drags the surrounding fluid, resulting in AC electro-osmotic flow. The magnitude of this flow depends on factors such as AC voltage, frequency, electrolyte conductivity, electrode geometry, and EDL thickness. At low to moderate AC frequencies (100 Hz to a few kHz), ACEO-driven flows dominate and can significantly enhance analyte transport in microfluidic biosensors. By directing biomolecules and nanoparticles toward the sensing region, ACEO improves detection sensitivity and reaction kinetics [43,44]. The velocity of ACEO flow (*u_ACEO_*) is expressed as follows:

$$u_{ACEO}\approx -\frac{\varepsilon_{m}}{\eta }\cdot \xi \cdot E_{t},$$

where *ε_m_* is the permittivity of the solution, *η* is the fluid viscosity, *ξ* is the zeta potential at the electrode interface and *E_t_* is the parallel component of the applied electric field. However, ACEO is strongest in low-conductivity solutions (<0.085 S/m) and becomes less effective as the conductivity increases [45]. Since the current study employs 0.05X PBS (≈0.08 S/m), which is close to this threshold, ACEO effects may still contribute slightly to fluid motion but are expected to be weak compared to ACET. AC electrothermal (ACET) flow, in contrast, arises due to temperature gradients generated by the non-uniform electric field at the electrode surface. These temperature variations lead to changes in the solution’s conductivity and permittivity, creating a convective flow that enhances the movement of H_2_O_2_ molecules toward the electrode surface [46]. The velocity of ACET-driven fluid motion (*u_ACET_*) is given as follows:

$$u_{ACET}\approx 5\times 10^{-4}\times \frac{\varepsilon_{m}\sigma V^{4}}{k\eta r}\left|\frac{1}{\sigma }\frac{\partial \sigma }{\partial T}\right|$$

where *V* is the applied voltage amplitude, *r* is the distance between adjacent electrode fingers, which was equal to 50 μm in this study, *T* is the absolute temperature, and *k* is the thermal conductivity of the solution. Since ACET flow is enhanced in solutions with higher conductivity, it plays a role in this study. Both ACEO and ACET effects facilitate the efficient transport of H_2_O_2_ molecules toward the Cu-Cys-GSH/Nafion-modified IDE sensor, enhancing detection sensitivity and reducing response time.

## 4. Results and Discussion

### 4.1. Sensor Characterization

**Figure 2:**
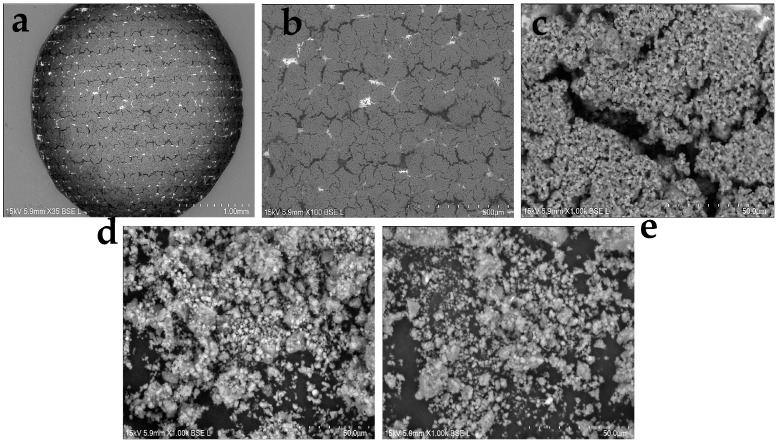
SEM images of (**a**–**c**) the electrode surface covered Cu-Cys-GSH/Nafion film at different scales, (**d**) Cu-L-Cys-GSH nanoparticles, and (**e**) Cu-D-Cys-GSH nanoparticles.

The surface morphology of the Cu-Cys-GSH nanoparticle–functionalized sensor and the synthesized Cu-L-Cys-GSH and Cu-D-Cys-GSH nanoparticles was analyzed using scanning electron microscopy (SEM), as shown in Figure 2. The low-magnification SEM image (Figure 2a, ×35) shows that the entire gold IDE sensor surface is uniformly coated with the Cu-Cys-GSH/Nafion film, confirming the successful surface modification via drop-casting. At medium magnification (Figure 2b, ×100), a network-like microstructure is observed, indicating the formation of interconnected nanoparticle aggregates within the Nafion matrix. The highest magnification (Figure 2c, ×1000) reveals a porous and granular texture, characteristic of nanostructured surfaces, where densely packed nanoparticles contribute to a high surface-to-volume ratio. This hierarchical nanostructure plays a crucial role in facilitating Fenton-like catalytic activity, enhancing charge redistribution, and improving the capacitive sensing response. Figure 2d,e reveals that both nanoparticle variants exhibit a granular and aggregated structure, suggesting successful self-assembly. The overall morphology appears similar between Cu-L-Cys-GSH and Cu-D-Cys-GSH, with no visually distinct differences in particle distribution or surface texture.

**Figure 3:**
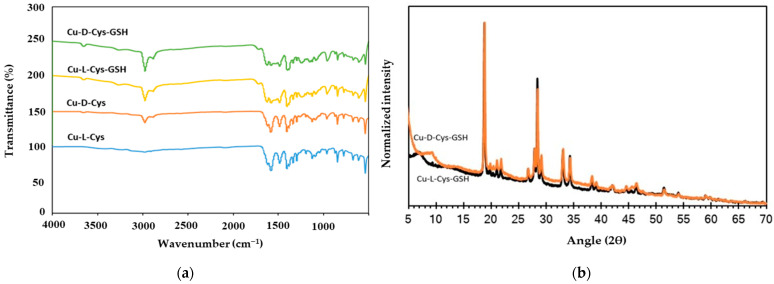
(**a**) FTIR spectra of synthesized chiral nanoparticles and (**b**) XRD patterns of Cu-L-Cys-GSH and Cu-D-Cys-GSH nanoparticles.

The FTIR spectra of Cu-L-Cys, Cu-D-Cys, Cu-L-Cys-GSH, and Cu-D-Cys-GSH nanoparticles, as shown in Figure 3a, confirm the successful interaction of cysteine and glutathione with copper through characteristic functional group vibrations. The broad absorption bands observed around 3200–3500 cm⁻^1^ correspond to O-H and N-H stretching vibrations, indicating the presence of hydroxyl and amine groups, which contribute to metal coordination. The peaks in the 1600–1700 cm⁻^1^ range are attributed to the C=O stretching of carboxyl groups, confirming metal-carboxylate interactions, which are common in amino acid–functionalized copper nanoparticles [47]. The disappearance of the thiol (-SH) peak around 2550–2600 cm⁻^1^ indicates that sulfur binds to copper through the formation of a Cu-S bond [29]. The peak around 611 cm⁻^1^ in Cu-Cys-GSH samples corresponds to Cu-S stretching vibrations, further confirming Cu-thiol interactions [29]. The additional peaks in the 1000–1200 cm⁻^1^ region correspond to C-N stretching from amine groups and asymmetric vibrations of C-O-C bonds, which indicate strong metal-ligand coordination with glutathione. The spectral differences between Cu-L-Cys-GSH and Cu-D-Cys-GSH suggest potential variations in molecular interactions due to chirality, affecting the self-assembly behavior of these nanoparticles. These findings confirm the formation of well-defined Cu-Cys and Cu-Cys-GSH nanoparticles with strong Cu-ligand interactions, validating their structural integrity.

X-ray diffraction (XRD) patterns shown in Figure 3b provide structural insights into the crystalline and amorphous phases of Cu-Cys-GSH nanoparticles. The presence of sharp diffraction peaks at 18.84°, 28.4°, and 34.4° indicates the characteristic diffraction planes associated with the incorporation of cysteine in the nanoparticle structure [29]. Additionally, the peaks observed around 29.2° suggest the formation of Cu(II)-thiolate complexes, confirming the coordination of copper with sulfur-containing ligands. The broad background signal at lower angles suggests the presence of amorphous regions, which may be attributed to the self-assembled nanostructure of Cu-Cys-GSH.

### 4.2. Optimization of Measurement Conditions

**Figure 4:**
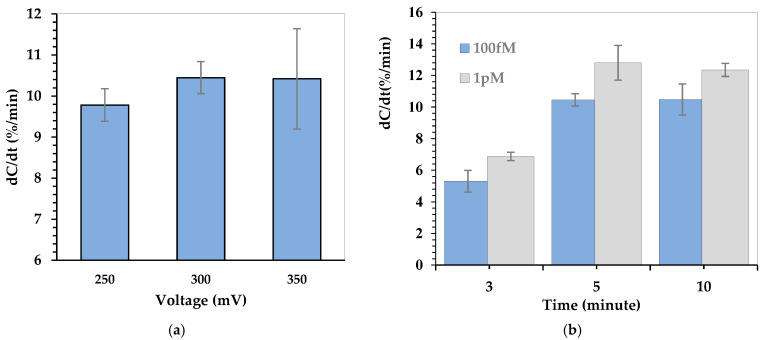
Normalized capacitance response for (**a**) applied voltage optimization and (**b**) incubation time optimization.

To ensure optimal performance of the Cu-L-Cys-GSH nanoparticle–functionalized capacitive biosensor, the effects of applied AC voltage and incubation time were investigated systematically. Figure 4 presents the capacitance change rate (dC/dt, %/min) under different testing conditions to determine the optimum voltage amplitude and incubation duration for reliable H_2_O_2_ detection. Figure 4a illustrates the effect of applied voltage (250, 300, and 350 mV) on the capacitance change rate (dC/dt, %/min). The influence of AC voltage on the sensor’s capacitance response was evaluated at a fixed frequency of 1 kHz with an H_2_O_2_ concentration of 100 fM. The results show that increasing the applied voltage from 250 mV to 300 mV enhances the capacitance response, suggesting improved charge redistribution and analyte interaction due to ACEK effects. However, at 350 mV, the variation in the signal increases, indicating possible instability at higher voltages. Based on these results, 300 mV was selected as the optimal voltage for further measurements. Figure 4b illustrates the effect of incubation time on the capacitance change rate (dC/dt, %/min) at two different H_2_O_2_ concentrations (100 fM and 1 pM). The results show that as the incubation time increases, the capacitance response also increases, indicating that a longer reaction time allows for more effective interaction between H_2_O_2_ and the Cu-L-Cys-GSH/Nafion film–modified sensor surface. The sensor response reaches a peak at 5 min, suggesting that sufficient reaction has occurred to induce a stable capacitive signal. Extending the incubation time does not significantly enhance the response. This behavior suggests that after 5 min, the interaction between H_2_O_2_ and the sensor surface stabilizes, and additional time may only lead to signal saturation rather than increased sensitivity.

### 4.3. Dose Response of Cu-Cys-GSH Nanoparticle–Functionalized Sensor

**Figure 5:**
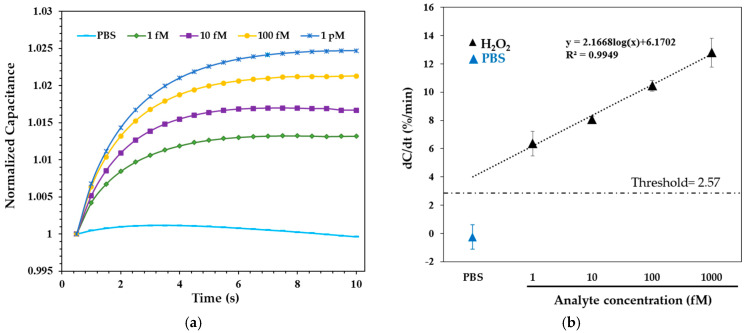
Capacitance response of H_2_O_2_ in 0.05X PBS. (**a**) Transient capacitance curves over 10 s. (**b**) Normalized dose-dependent response. Data are presented as mean ± SD from three independent replicates. Measurements were performed at 300 mV and 1 kHz.

H_2_O_2_ solutions ranging from 1 fM to 1 pM in 0.05X PBS were applied to the Cu-L-Cys-GSH/Nafion film–modified electrode surface and incubated for 5 min before measurement. Following the incubation, the normalized capacitance was recorded for 10 s under the optimized AC conditions. Figure 5a presents the normalized capacitance-time profiles following incubation with H_2_O_2_. A concentration-dependent increase in capacitance was observed, where higher H_2_O_2_ levels led to a more rapid and pronounced rise within the 10-s measurement window. This trend is attributed to enhanced charge accumulation at the sensor interface, driven by the Fenton-like reaction between H_2_O_2_ and Cu-Cys-GSH nanoparticles.

Figure 5b shows the corresponding dose-response curve, where the capacitance change rate (dC/dt, %/min) is plotted against H_2_O_2_ concentrations ranging from 1 fM to 1 pM, with logarithmic scaling applied in the data fitting. The sensor exhibited excellent linearity across this range, following the equation dC/dt = 2.1668 log_10_(x) + 6.1702, with a correlation coefficient (R^2^) of 0.9949, where x is the H_2_O_2_ concentration in femtomolar (fM). The detection threshold, calculated as three times the standard deviation of the blank [32], was determined to be 2.57%/min. Substituting this value into the dose-response equation yielded a limit of detection (LOD) of 21.8 aM. These results establish the Cu-Cys-GSH nanoparticle–functionalized capacitive biosensor as a highly sensitive and stable system for H_2_O_2_ detection.

### 4.4. Influence of Chirality on Cu-Cys-GSH Nanoparticle–Functionalized Sensor Response

**Figure 6:**
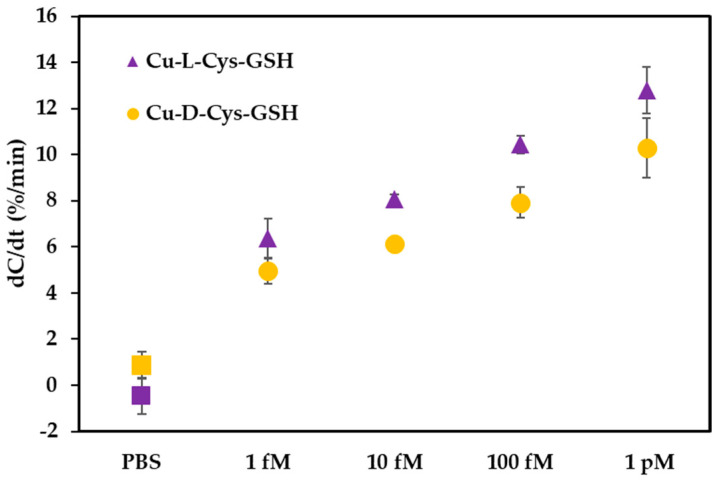
Influence of chirality on the capacitive response (dC/dt, %/min) of sensors functionalized with Cu-L-Cys-GSH and Cu-D-Cys-GSH nanoparticles across varying concentrations of H_2_O_2_ (1 fM to 1 pM). The data indicate enhanced sensor sensitivity for the L-enantiomer nanoparticles compared to the D-enantiomer counterparts.

To investigate the influence of chirality on sensor performance, we evaluated the capacitive responses of sensors functionalized with Cu-L-Cys-GSH and Cu-D-Cys-GSH nanoparticles across a range of H_2_O_2_ concentrations (Figure 6). The experimental results indicate that the sensor modified with Cu-L-Cys-GSH nanoparticles consistently outperformed the sensor modified with Cu-D-Cys-GSH nanoparticles at all tested concentrations, ranging from 1 fM to 1 pM. Specifically, Cu-L-Cys-GSH nanoparticles exhibited higher sensitivity (greater dC/dt values) compared to their D-enantiomer counterparts.

This difference in performance can be attributed to distinct chiral interactions between the nanoparticles and endogenous biomolecules such as glutathione. As supported by previous research [29], Cu-L-Cys nanoparticles demonstrated a higher affinity toward L-GSH due to a more energetically favorable binding process, confirmed by isothermal titration calorimetry and molecular dynamics simulations. The stronger affinity between L-enantiomer nanoparticles and L-GSH accelerates the redox reaction between them, significantly enhancing the catalytic efficiency of the subsequent Fenton-like reaction [29]. The redox conversion of Cu(II) to Cu(I) is facilitated by more efficient electron transfer. Specifically, tighter molecular interactions reduce the electron transfer distance and lower the activation energy barrier. Additionally, stronger binding creates a localized high concentration of L-GSH at the nanoparticle surface, further enhancing the redox cycling rate. This improved electron transfer efficiency ultimately strengthens the catalytic performance of the Fenton-like reaction. The increased catalytic activity of Cu-L-Cys-GSH nanoparticles results in enhanced generation of hydroxyl radicals (·OH), thereby amplifying the sensor’s response through changes in capacitance, driven by intensified local redox cycling and shifts in conductivity.

### 4.5. Selectivity

Selectivity is a key parameter in the development of biosensors, particularly when applied to complex biological environments containing diverse interfering species. To evaluate the selectivity of the Cu-L-Cys-GSH/Nafion-functionalized capacitive sensor, potential interferents such as amino acids (L-alanine and L-aspartic acid) and the electroactive neurotransmitter dopamine were selected.

**Figure 7:**
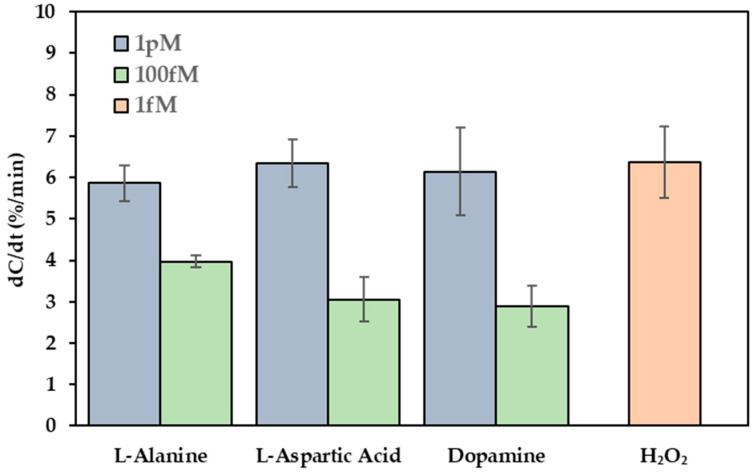
Selectivity of the Cu-L-Cys-GSH/Nafion-functionalized sensor. Sensor responses toward H_2_O_2_ (1 fM) and potential interfering species (L-alanine, L-aspartic acid, and dopamine) at concentrations of 100 fM and 1 pM in 0.05X PBS.

Figure 7 presents the selectivity performance of the sensor when exposed to these interferents in comparison to H_2_O_2_. The non-target species were tested at concentrations of 100 fM and 1 pM using an AC signal of 1 kHz and 300 mV. All non-target species exhibited lower responses than that of 1 fM H_2_O_2_ under identical conditions. Specifically, at a concentration of 1 fM, H_2_O_2_ yielded a capacitance change rate (dC/dt) of 6.36 ± 0.865%/min, while L-alanine, L-aspartic acid, and dopamine at 1 pM exhibited dC/dt values of 5.86 ± 0.43%/min, 6.35 ± 0.57%/min, and 6.14 ± 1.065%/min, respectively. Therefore, the sensor demonstrates good specificity for H_2_O_2_, with a selectivity of approximately 1000:1. The low standard deviation values further support the reproducibility across different electrodes.

The significant discrimination between H_2_O_2_ and the interfering species can be attributed to the catalytic specificity of the Cu-L-Cys-GSH nanoparticles, which facilitated a Fenton-like reaction exclusively with H_2_O_2_, resulting in amplified charge redistribution and enhanced capacitive transduction. In contrast, the non-catalytic species, including amino acids and dopamine, likely induced only minor surface perturbations or weak, non-specific adsorption, and a substantially attenuated capacitance response.

### 4.6. Sample Analysis

The reliability of the Cu-L-Cys-GSH nanoparticle–functionalized capacitive biosensor was validated through the analysis of spiked milk samples. As presented in Table 1, the sensor exhibited excellent recovery rates, ranging from 109.2% to 126.2% in milk across H_2_O_2_ concentrations of 10 fM, 100 fM, and 1 pM. The relative standard deviation (RSD) values remained consistently low (<0.6%), underscoring the high precision of the sensor in complex matrices. Saliva testing data are included in the Supplementary Materials.

These results confirm the sensor’s capability for accurate and consistent detection of trace levels of H_2_O_2_ in real samples, demonstrating its robustness and applicability for practical diagnostic applications.

## 5. Conclusions

In this study, we developed a capacitive biosensor functionalized with Cu-Cys-GSH nanoparticles for highly sensitive detection of H_2_O_2_. The detection mechanism is based on a Fenton-like reaction, where redox cycling between Cu^2+^ and Cu^+^ generates hydroxyl radicals (·OH), causing localized alterations in electrolyte composition, ionic mobility, and charge distribution at the electrode interface. The influence of nanoparticle chirality was also explored, demonstrating that sensors modified with Cu-L-Cys-GSH nanoparticles exhibited superior capacitive responses compared to the D-enantiomer. This difference can be attributed to stereochemistry-dependent interactions with glutathione, influencing catalytic efficiency. Analysis using spiked milk demonstrated the sensor’s high accuracy and precision. The findings confirm the effectiveness of Cu-Cys-GSH nanoparticles in enhancing capacitive biosensor performance through chirality-dependent catalytic mechanisms. Based on these results, the sensing platform holds potential for extension toward detecting diverse biological and chemical targets, particularly redox-active species, for future applications in clinical diagnostics, food safety analysis, and environmental monitoring.

## Figures and Tables

**Table 1:** Detection of H_2_O_2_ in milk samples using Cu-L-Cys-GSH/Nafion film–modified sensor.

Samples	Spiked/fM	Found/fM	Recovery/%	RSD/%
1	10	10.92	109.2	0.37
2	100	120	120	0.57
3	1000	1262	126.2	0.35

## Data Availability

The original contributions presented in this study are included in the article. Further inquiries can be directed to the corresponding author.

## Supplementary Materials

The following supporting information can be downloaded at: https://www.mdpi.com/article/10.3390/bios15050315/s1, Table S1: Detection of H_2_O_2_ in saliva samples using Cu-L-Cys-GSH/Nafion film-modified sensor.
