# Supplementary material for: Chiral-Dependent Redox Capacitive Biosensor Using Cu-Cys-GSH Nanoparticles for Ultrasensitive H2O2 Detection
Source: Biosensors (Basel). 2025 May 14;15(5):315. doi: 10.3390/bios15050315 (PMC12110360; doi:10.3390/bios15050315)
Supplement: Supplementary file 1 [file biosensors-15-00315-s001.zip › biosensors-3582820-supplementary.pdf]

Chiral-Dependent Redox Capacitive Biosensor Using Cu-Cys-GSH Nanoparticles for Ultrasensitive H_2O_2 Detection

Duygu Yilmaz Aydin [1,2], Jie Jayne Wu[1*], Jiangang Chen[3]

[1]Department of Electrical Engineering and Computer Science, The University of Tennessee, Knoxville, TN 37996, USA

[2]Department of Bioengineering, Malatya Turgut Ozal University, 44210, Malatya, Türkiye

[3]Department of Public Health, The University of Tennessee, Knoxville, TN 37996, USA

*Correspondence: jwu10@tennessee.edu

Materials and Methods

Sample preparation

To evaluate the applicability of the developed biosensor, H_2O_2 detection was also performed in saliva. The saliva samples were collected into sterile tubes and immediately stored at 4 °C. The samples were centrifuged at 4000 rpm for 10 minutes to remove debris and particulates. The resulting clear supernatant was diluted 10 times with 0.05X PBS.

Results and Discussion

Sample Analysis

The reliability of the Cu-Cys-GSH nanoparticle-functionalized capacitive biosensor was validated through the analysis of spiked saliva samples. As presented in Table S1, the sensor exhibited excellent recovery rates, ranging from 96% to 105% in saliva across H_2O_2 concentrations of 10 fM, 100 fM, and 1 pM. The low relative standard deviation (RSD) values (<0.8%) highlight the excellent precision and reliability of the sensor when applied to complex matrices.

These results confirm the sensor's capability for accurate and consistent detection of trace levels of H_2O_2 in real-world biological samples, demonstrating its robustness and applicability for practical diagnostic applications.

Table S1. Detection of H_2O_2 in saliva samples using Cu-L-Cys-GSH/Nafion film-modified sensor.

Samples	Spiked/fM	Found/fM	Recovery/%	RSD/%
1	10	9.6	96	0.1
2	100	98.5	98.5	0.76
3	1000	1050	105	0.20
